# How does protein aggregate structure affect mechanisms of disaggregation?

**DOI:** 10.1042/BST20253077

**Published:** 2025-07-28

**Authors:** YuChen Yang, Hays S. Rye

**Affiliations:** Department of Biochemistry and Biophysics, Texas A&M University, College Station Texas, 77845, U.S.A.

**Keywords:** amyloid, molecular chaperones, proteostasis, protein aggregation, protein misfolding

## Abstract

Protein misfolding and aggregation underpin numerous pathological conditions, including Alzheimer’s, Parkinson’s, and Huntington’s diseases. Within cells, the competition between protein folding and misfolding-driven aggregation necessitates intricate quality control systems known collectively as the proteostasis network, with molecular chaperones playing central roles. Critical gaps remain in our understanding of why certain protein aggregates are amenable to efficient chaperone-mediated disassembly, while others resist such intervention. Aggregates can be most broadly categorized into structurally ordered amyloid fibrils and more irregular amorphous clusters. Amyloid fibrils are characterized by a highly structured, cross-β-sheet architecture, and they generally display nucleation-driven growth kinetics. In contrast, amorphous aggregates form through heterogeneous interactions among partially unfolded proteins, which typically lack ordered and repeating structure but still display poorly understood, specific assembly constraints. Importantly, amorphous aggregation and amyloid formation are often linked to one another, with several different types of aggregate structures forming at the same time. The ability of molecular chaperones to remodel and disassemble aggregates is affected by aggregate size, internal structure, surface dynamics, and exposure of chaperone-binding sites. However, despite these insights, the mechanistic complexity, aggregate heterogeneity, and dynamic properties present substantial experimental and theoretical challenges. Addressing these challenges will require innovative approaches combining single-molecule biophysics, structural biology, and computational modeling to unveil universal principles governing protein aggregation and disaggregation within cellular environments.

## Introduction

The folding of proteins within the complex and concentrated interior of a cell frequently goes awry, resulting in misfolding and aggregation [1,2]. Work on a variety of human diseases has shown that the incorrect folding and/or aggregation of important cellular proteins can lead to serious pathologies [3,4]. Misfolded proteins can generate infectious particles that propagate their own formation upon spreading from cell to cell [5,6], and aggregation hinders the production of medically and industrially important proteins [7,8]. While the deleterious consequences of protein misfolding and aggregation have been recognized for some time, recent work has shown that reversible protein aggregation also plays important roles in basic cellular physiology [9]. For example, dynamic aggregation is essential for the collection and concentration of biomolecules during cell stress and appears to play a role in gene regulation during cell division [10–13].

The competition between productive folding and pathological aggregation, along with the need to control functional protein aggregation, led to the evolution of specialized protein folding machines, known as molecular chaperones, which are required for normal protein biogenesis and homeostasis [14]. These chaperone families are both ancient and highly conserved, in both structure and function [15]. In addition to facilitating productive *de novo* folding, several molecular chaperone families are also essential for the cellular response to aggregation, recognizing and working to dismantle aggregate particles [16]. These chaperones also function in partnership with regulated, chambered proteases like the proteasome and, in eukaryotic cells, with elaborate salvage pathways that involve membrane-enclosed degradation chambers like the lysosome [17]. In total, these systems of protein surveillance and quality control are referred to as the cellular proteostasis network [18]. However, why some protein aggregates are quickly dismantled and their constituent monomers efficiently refolded or degraded, while others are almost completely refractory to disaggregation remains poorly understood.

Developing a more complete picture of protein aggregation and disaggregation faces a profound challenge: the molecular details of protein aggregation are highly complex. Different proteins can display radically distinct aggregation behavior that is, fundamentally, governed by poorly understood features of non-native folding intermediates and how they interact with one another. Even for a single protein, modest differences in environmental conditions can produce distinctive aggregate types that can display highly varied susceptibilities to chaperone-mediated disassembly. Multiple aggregate types can emerge at the same time, with distinctive conformational features, growth kinetics, and internal stabilities, all of which can change across a range of time scales. Capturing the key mechanistic details, without unintentionally altering the underlying physical events through experimental manipulation or creating models that are overly biased by the mean behavior of these heterogeneous nanoparticle samples, is a substantial challenge. Here, we review some of the recent progress in understanding features of different types of protein aggregates and how they affect disassembly by molecular chaperones.

## Amorphous aggregates, amyloids, and condensates

**Figure 1:**
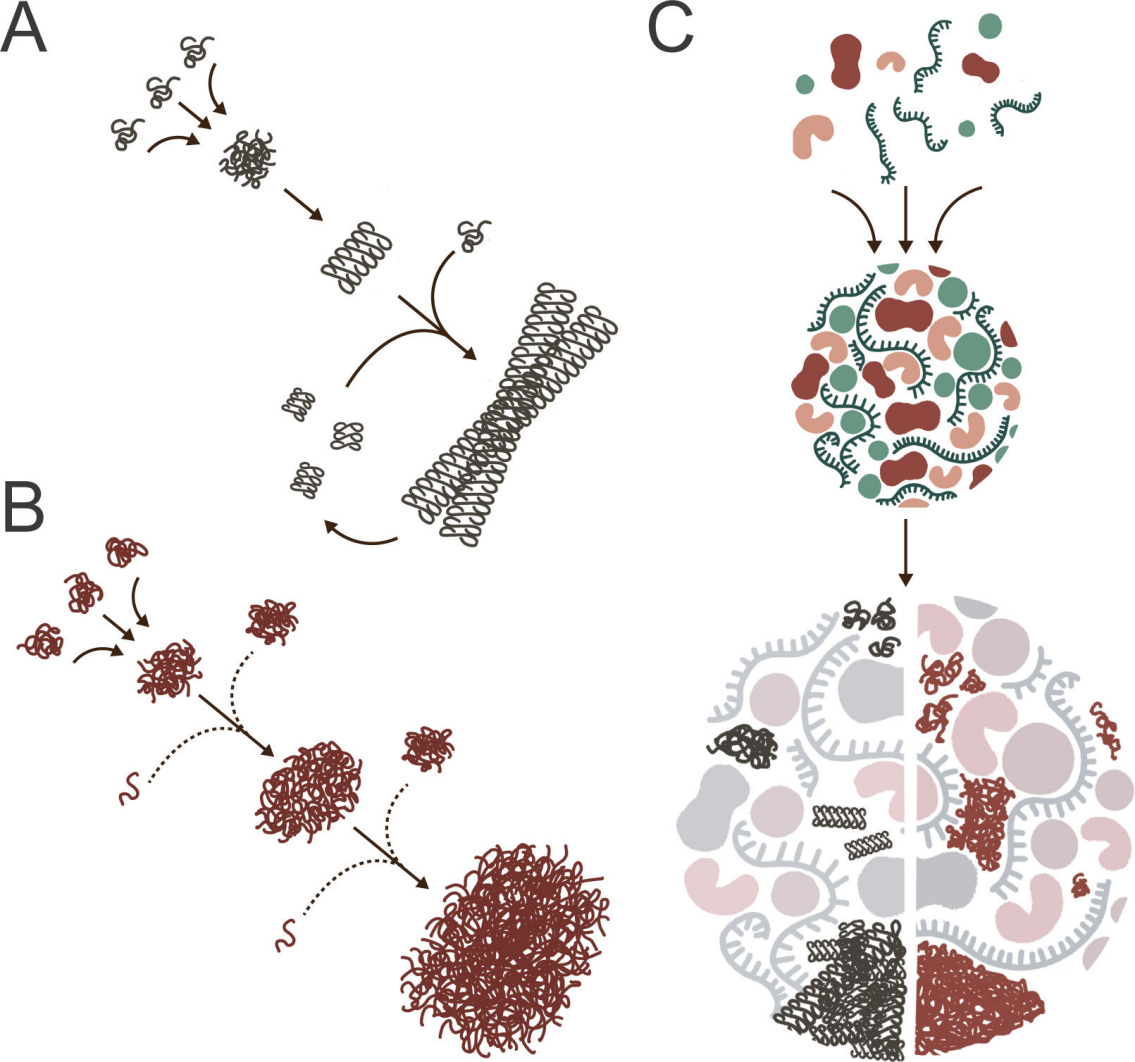
Formation of aggregates, amyloids, and biomolecular condensates (**A**) Amyloid fibril formation typically involves a rate-limiting initiation phase, where misfolded proteins form a stable, amorphous, or partially structured cluster. Internal rearrangements create small, oligomeric nuclei that are enriched in extended β-sheet structures, which act as polymerization seeds. Directional addition of monomers to the initiating seeds through complementary β-sheet stacking creates protofibrils that elongate, eventually becoming large enough to interact with other protofibrils and form mature fibrils. Fragmentation of existing fibrils and/or protofibrils accelerates amyloid formation by creating new ends and seeds. (**B**) Amorphous aggregates generally form through low-specificity protein clustering interactions, frequently involving non-native hydrophobic contact between monomers. Distinct from amyloids, amorphous aggregates generally lack regular or repeating overall structure. While amorphous aggregate formation often involves the misfolding or unfolding of monomers, aggregating subunits need not be devoid of defined secondary or even tertiary structure and can grow through expansive and highly stable domain-swapping networks. (**C**) Biomolecular condensates arise through the phenomenon of liquid–liquid phase separation (LLPS), which is driven by weak, highly dynamic multivalent interactions among proteins and, often, nucleic acids. In some cases, condensates play important biological roles by segregating biomolecules into distinct cellular sub-domains as membrane-less compartments. In other cases, LLPS separation appears to serve as an initial concentrating event, which then leads on to either, or both, amorphous aggregation and amyloid formation. In the figure, a condensate droplet is depicted as separating into two halves, representing potential alternative pathways of aggregate formation during condensate maturation. Sub-domain formation within the condensate can create regions with locally high concentrations of misfolded or aggregation-prone proteins, which can promote the accumulation of either amorphous aggregates (right half) or amyloid aggregates (left half). Notably, one pathway may predominate, or both may coexist within the same condensate, depending on cellular context and biophysical conditions.

Protein aggregates can be loosely categorized by their mesoscale morphology and the extent to which this morphology is dominated by well-organized, repeating structure [19]. In the most general terms, aggregates tend to display either extended, fibril-like shapes or poorly defined, irregular, and amorphous geometries. Fibrillar aggregates can form via aberrant assembly of otherwise well-folded and native proteins, as in the case of sickle cell hemoglobin at low oxygen tension [20], or from structurally reorganized monomers, which coalesce into extended, cross-β-sheet, super-helices known as amyloids (Figure 1A) [21–23]. Amyloid formation has been linked to a wide spectrum of diseases and appears to be particularly problematic in neural tissues, with disorders like Alzheimer’s, Parkinson’s, and Huntington’s disease, frontotemporal dementia, and prion diseases in humans, cows, sheep, deer, and other wild cervids—all linked to the pathological formation of amyloid protein fibrils [3,4].

Amorphous aggregates generally form through low-specificity protein clustering interactions, frequently involving non-native hydrophobic contact between monomers (Figure 1B) [24–26]. Distinct from amyloids, amorphous aggregates generally lack regular or repeating overall structure [27,28]. While amorphous aggregate formation often involves the misfolding or unfolding of monomers, aggregating subunits need not be devoid of defined secondary or even tertiary structure and can grow through expansive and highly stable domain-swapping networks [24,26,29]. For example, high-level expression of proteins in bacteria often results in extensive amorphous aggregation and, in some cases, amyloid-like aggregate formation [30,31]. These aggregates tend to pack into electron-dense structures at the poles of the cell and are referred to as inclusion bodies [32]. Compared with classical amyloid fibrils, amorphous aggregates and bacterial inclusion bodies are more readily disrupted by mildly denaturing conditions and are typically more susceptible to proteolysis, suggesting looser packing interactions and reduced structural order at the molecular level [33–36]. However, some amorphous aggregates and inclusion bodies display high protease resistance, suggesting that they possess substantial local order, are tightly packed, or possess other structural features that result in proteolytic shielding [37,38]. These observations further underscore the structural heterogeneity of amorphous assemblies.

Importantly, the classification boundary between amyloid and amorphous aggregates is poorly defined [19,33]. While amyloids are recognized by their characteristic cross-β structure, some aggregates, which do not form canonical amyloid fibrils, share similar pathological and biophysical features, like enriched β-sheet content and increased protease resistance. These aggregates are often classified as ‘amyloid-like’ [33]. Additionally, some proteins appear to interconvert between more amyloid-like and amorphous forms, and amorphous aggregates have been suggested to be an early intermediate in the sequence of events that leads to amyloid fibril formation for many proteins [23]. Both amorphous and amyloid aggregates display critical assembly behavior, where aggregate formation cannot occur below a minimal concentration threshold [39]. However, the detailed assembly of amyloid and amorphous aggregates is typically distinct. Amorphous aggregates can form in a variety of different ways, incorporating partially folded, misfolded, and even unfolded monomer forms (Figure 1B) [24,26,29]. Monomer assembly into amorphous aggregate particles appears to be limited only by the exposed interaction valence of the aggregating monomers and the multi-subunit particles they form. Under some conditions, amorphous aggregates appear to form through a process reminiscent of a glass-like phase transition [25]. By contrast, amyloid formation generally follows nucleation-polymerization behavior, in which the rate-limiting step for amyloid growth involves the low-probability formation of an ordered amyloid seed [40,41]. Seeds provide the growth platform for a polymerization reaction that extends the ends of the protofibril, as monomers are added to the growing amyloid β-sheet (Figure 1A) [40,42,43]. As they grow, protofibrils tend to break into smaller pieces, either through the accumulation of mechanical strain, the action of molecular chaperones, or other quality control events [44–49]. Fibril severing exposes new ends, which become new growth sites. The resulting progressive amplification, in some cases stimulated by surface binding and secondary nucleation events along the sides of growing fibrils, leads to the explosive formation of structured amyloid fibrils [23].

The subunit contacts that mediate amorphous aggregate growth are thought to be much less dependent on specific structural features than those of amyloid fibrils. For example, distinct structural isoforms of several disease-associated amyloids have been identified, which cannot co-assemble [50–53]. These structural polymorphisms appear to enforce a high level of assembly specificity, which prevents even subtly distinct conformational variants from interacting with one another, a phenomenon thought to underpin the observation of species-specific prion strains [54]. Yet, despite their much greater conformational variation, assembly heterogeneity, and morphological diversity, formation of amorphous aggregates is nonetheless influenced by structural constraints. For example, it is uncommon for different, amorphously aggregating proteins to co-assemble, suggesting that the more loosely structured non-native monomers that form amorphous aggregates also possess hidden conformational specificity that limits how they grow [55,56]. Consistent with this suggestion, we recently observed that two populations of amorphously aggregating *R. ruburm* RuBisCO, which are separated by less than a minute of aggregate growth time, are 4–6 times less prone to form co-aggregate particles than they are with themselves [57].

Recent work on bimolecular condensates suggests that connections between local protein concentrations, amorphous aggregation, and amyloid seed formation may be more complex in a living cell than previously imagined (Figure 1C) [58–60]. Biomolecular condensates arise through the phenomenon of liquid–liquid phase separation (LLPS), which is driven by weak, highly dynamic multivalent interactions among proteins and, often, nucleic acids [59,61]. Functional condensates like stress granules and P-bodies provide membrane-less intracellular assembly domains for various biomolecules in response to cellular stress [60]. However, the formation of LLPS domains may also serve as an initiating and concentrating event that leads to either, or both, amorphous aggregation and amyloid formation (Figure 1C) [59,60]. For example, studies of several disease-associated proteins, such as FUS, TDP-43, α-synuclein, Aβ-42, and Tau, have suggested that condensate formation precedes both aggregation and amyloid formation [62–66]. During condensate formation, distinct sub-domains can emerge within the LLPS phase, which can be populated by proteins enriched in particular conformations or that facilitate local increases in bimolecular concentration [67]. If the characteristics of these sub-domains favor recruitment of incorrectly folded intermediates or conformers prone to aggregation, they can act as nucleating zones that dramatically accelerate the formation of amyloids and amorphous aggregates [65,68,69].

## Chaperone-mediated disaggregation

Because the early response to protein aggregation is centered on a subset of the cellular molecular chaperone machinery, we briefly outline some of the features of these systems. More detailed reviews, including descriptions of their structures, mechanisms, and broader activities, can be found elsewhere [70–76].

### Small heat shock proteins

**Figure 2:**
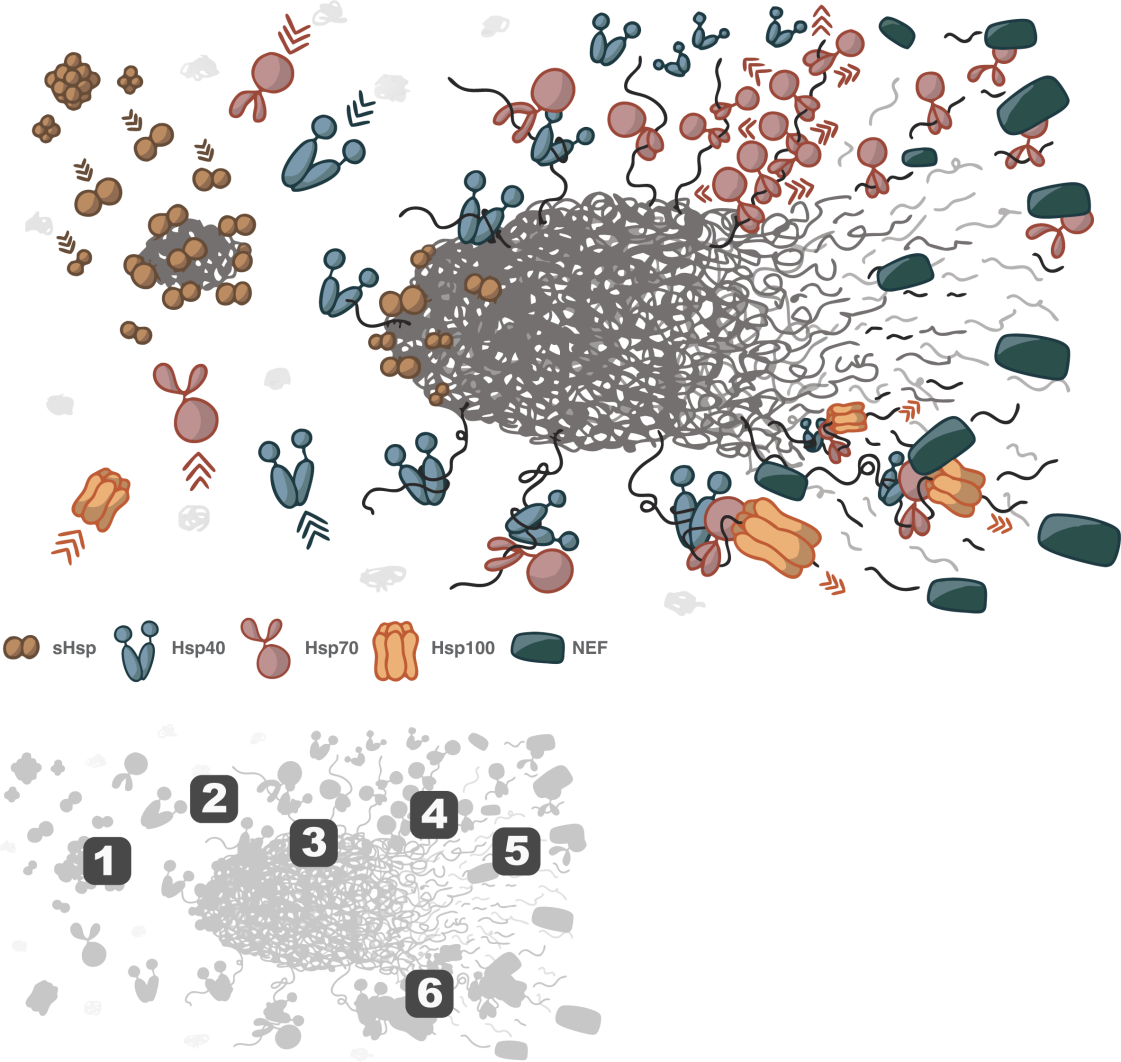
Chaperone-mediated disaggregation Protein disaggregation is a complex, multi-step process, involving several families of molecular chaperones. While the details of aggregate disassembly depend on the specific features of each aggregate particle, several features appear to be widely shared. The key provides a guide to some of the common properties illustrated in the main figure. (1) Stress-induced aggregation is typically associated with early intervention by small heat shock proteins (sHsps), which undergo stress-induced de-oligomerization to release active dimeric species that bind to aggregating proteins, halting further aggregate growth and priming the aggregates for action by the next set of chaperones. (2) Hsp40s are thought to initially recognize the aggregate surface and recruit ATP-bound Hsp70s. (3) Formation of an Hsp40-Hsp70 complex triggers an ATP hydrolysis-dependent conformational change in the Hsp70s, enabling them to tightly bind exposed polypeptide segments at the surface of the aggregate. (4) Repeated recruitment of Hsp40s and Hsp70s has been proposed to facilitate the formation of spatially localized Hsp70 clusters, which are thought to create an unfavorable, restricted conformational entropy for the closely packed molecular chaperones. Minimizing this unfavorable energetic condition has been theorized to provide a disruptive pulling force away from the aggregate surface. (5) Nucleotide exchange factors (NEFs) bind to the Hsp70s, facilitating release of the Hsp70 from the substrate protein. (6) In eubacteria, fungi, and plants, Hsp100s also assist in the force-dependent disruption of aggregates. Hsp100s are members of the AAA+ family of ATP-driven extrusion motors, which are recruited by the Hsp70-substrate complex to the aggregate surface. Transfer of the substrate protein to the Hsp100 results in the ATP-dependent threading of the protein through the Hsp100’s central channel, providing an additional, directional pulling force.

The small heat shock proteins (sHsp) are ATP-independent chaperones that serve as the cellular first line of defense against protein aggregation, binding early aggregation intermediates and arresting their growth [15,77,78]. The sHsps are particularly important during systemic environmental stresses, such as elevated temperatures, which can induce wide-scale protein unfolding [70–72]. Structurally, most sHsps possess the same overall monomer organization: a highly conserved α-crystallin domain (ACD), an unstructured N-terminal domain (NTD), and an unstructured C-terminal domain (CTD) [72,79]. Under non-stress conditions, sHsps generally self-assemble into inactive, polydisperse, dynamic oligomers, with their client protein-binding sites buried within the oligomer [80–82]. The application of unfolding stress induces spontaneous disassembly of these inactive oligomers, liberating monomers and dimers and exposing their client protein binding sites, which then target non-native, aggregating proteins (Figure 2) [70,83,84]. Importantly, sHsps do not possess intrinsic disaggregase activity. Rather, they act as ‘holdase’ systems that bind and block exposed, aggregation-driving surfaces found on non-native proteins, thereby inhibiting large-scale aggregate growth [85]. Disassembly of these sHsp-protein aggregate co-particles ultimately requires the action of other, ATP-utilizing molecular chaperones (Figure 2) [77,86–91].

Remarkably, sHsps appear to be capable of adapting how they interact with and inhibit different aggregating client proteins. For example, distinct binding behavior has been observed for some sHps when they interact with amyloid versus amorphous aggregates [92]. For amyloid fibrils, such as α-synuclein and tau, the hydrophobic groove within the ACD appears to play a critical role in substrate protein binding [93,94]. At low concentrations, the human sHsps αB-crystallin and Hsp27 transiently bind to the ends of apolipoprotein C-II amyloid fibrils and block fibril elongation [95,96]. However, at higher concentrations, binding of sHsps appears to expand to lateral surfaces of amyloid protofibrils, blocking the assembly of larger fibrils and/or secondary nucleation events [97]. By contrast, binding of sHsps to amorphous aggregates appears to depend more strongly on interactions between non-native client proteins and the flexible NTD and CTD domains [98,99]. At the same time, co-particles formed by different sHsps and amorphous aggregates display widely divergent physical properties. For example, many sHsps appear to form stable, heterogeneous, high molecular mass complexes that can be easily sedimented when interacting with non-native, aggregating forms of the model proteins lysozyme, luciferase, malate dehydrogenase, citrate synthase, and GFP [90,91,99–103]. By contrast, we have observed that the *E. coli* sHsps IbpAB form lower-mass co-particles containing only 20–40 non-native client proteins when interacting with amorphously aggregating forms of the bacterial prolidase enzyme PepQ and RuBisCO [104]. In another example, αB-crystallin appears to form complexes with both amyloid and amorphous forms of the model protein α-lactalbumin that are similar [105]. Importantly, while the molecular details of sHsp interaction with amorphous aggregates remain particularly obscure, their incorporation into co-particles dramatically facilitates subsequent aggregate disassembly and protein recovery [77,86–90].

### Hsp70s and co-chaperones

Sitting at the core of the cellular molecular chaperone network are the Hsp70s [73,74]. These ATP-powered machines are constructed from two primary domains: an amino-terminal nucleotide-binding domain (NBD) and a substrate-binding domain (SBD) [74]. The SBD is built from an open, curved β-sheet sub-domain that binds extended segments of polypeptide and is joined to an elongated α-helical lid sub-domain. The NBD and SBD are connected to one another by a flexible linker, which is essential for allosteric communication between the two domains [106]. Along with two co-chaperone families—the Hsp40s, which are members of the large and diverse J-domain protein superfamily, and nucleotide exchange factors (NEFs) like GrpE in bacteria and Hsp110s in eukaryotes—the Hsp70s act as ATP-regulated molecular clamps, binding and releasing linear segments of amino acids that are enriched in small hydrophobic amino acids, degenerate sequences that occur with moderate frequency in most proteins [73,74,107,108]. The Hsp70 cycle begins when an Hsp40 recognizes and binds a non-native, aggregating protein surface and recruits a monomer of Hsp70 [76]. Interactions between the Hsp40 and Hsp70 result in transfer of a segment of the non-native protein into the open Hsp70 SBD, which triggers hydrolysis of ATP and closure of the SBD lid, locking a piece of the client protein into the Hsp70 SBD clamp [109–111]. Opening of the SBD lid requires the post-hydrolysis exchange of ADP for ATP, a process that is catalyzed by a NEF [74]. Repeated rounds of this functional cycle result in the rapid loading and unloading of Hsp70s on the surface of aggregate particles (Figure 2) [46,112].

In some cases, the ATP-driven Hsp70 cycle can drive disassembly of protein aggregates [113–115]. In other cases, aggregates are refractory to disaggregation by the Hsp70s alone, unless sHsps are first incorporated into the aggregate particles [101,104,115–117]. Exactly how interactions between Hsp70s, their co-chaperones, aggregated monomers, and sHsps lead to effective disaggregation remains elusive, though several models have been proposed. In one model, termed ‘entropic pulling,’ recruitment of Hsp40s and Hsp70s has been suggested to create spatially localized Hsp70 clusters at the surface of an aggregate particle (Figure 2) [46,117–119]. Close apposition of the large, ~70 kDa Hsp70 monomers with one another, as well as the aggregate surface, results in dynamic restriction of the Hsp70-client protein complex and a locally unfavorable conformational entropy. Minimizing this unfavorable energetic condition is proposed to provide a disruptive pulling force that drives the bound Hsp70s, and the segments of the aggregated protein to which they are attached, away from the aggregate surface. Studies using the fibrillar form of α-synuclein appear consistent with this model [46,118]. The larger exposed client binding zones found at the ends of the α-synuclein fibril seem to facilitate clustering of Hsp70s in such a way that the larger Hsp110 NEF cannot access the Hsp70 NBD to facilitate nucleotide exchange and dissociation. The resulting unfavorable local concentration of bound Hsp70s has been estimated to generate a pulling force directed away from the fibril end of approximately 46 pN over distances of 1 nm [120]. Whether this disruptive action results in exclusive removal of monomers or fragmentation at the fibril ends, or both, remains unclear (Figure 2). Super-stoichiometric recruitment of Hsp70 to luciferase amorphous aggregates has also been observed and suggested to result in similar disruptive, entropic pulling forces [121]. However, the effectiveness of an entropic pulling device at the surface of an amorphous aggregate, with potentially much less regular and concentrated Hsp70 binding sites, and potentially much more dynamic and compliant surfaces, remains unclear.

### Hsp100/Hsp70 bi-chaperone system

Some aggregates, even in the presence of sHsps, remain resistant to Hsp70-mediated disassembly. In bacteria, fungi, and plants—but not, apparently, in metazoans—a second, specialized disaggregation system can be called upon [75,76,122]. Generally referred to as Hsp100s, these hexameric members of the AAA+ATPase superfamily function as ATP-powered extrusion machines [123–125]. Monomers of Hsp100, each of which contain two ATPase domains, assemble into an oligomer with a narrow, water-filled central pore in the presence of ATP [125–127]. Key insights into the structure and mechanics of the Hsp100s have been uncovered over the last two and a half decades, including how they cooperate with Hsp70s to create a so-called ‘bi-chaperone disaggregase’ [128–131]. Loading of an Hsp100 hexamer onto aggregates appears to first require binding of an Hsp70 to the aggregate surface, which is driven by the functional cycle outlined above (Figure 2) [132,133]. The ATPase rate of the Hsp100 hexamer and the rate at which it can thread an extended polypeptide through its central pore depend on the position of an extended helical coiled-coil middle domain located on the exterior of the Hsp100 cylinder [134–137]. Importantly, this α-helical domain also contains a specialized binding site for an Hsp70 [138]. The active Hsp70/Hsp100 bi-chaperone disaggregase system is thus thought to involve: (1) initial Hsp70-dependent recruitment of the Hsp100 hexamer to the aggregate surface, (2) transfer of the client protein from the Hsp70 to the Hsp100, and (3) activation of Hsp100 ATPase activity [75,76]. Displacement of the Hsp100 α-helical domains by Hsp70s serves a similar function as the throttle of a mechanical engine, activating a polypeptide threading motor that can locally generate pulling forces of up to ~50 pN, which are thought to be capable of extracting entire monomer subunits from an aggregate (Figure 2) [123].

The specific consequences of multiple Hsp100 and Hsp70 machines pulling on different parts of an aggregate particle, in different locations, remain poorly understood. One recent study [49], which examined the disaggregation of amyloid fibrils created from the prion-forming region of the yeast translational termination protein Sup35, observed two distinct disassembly pathways in the presence of the yeast bi-chaperone disaggregase system: fragmentation and monomeric dissolution. Specific features of the amyloid core regions and the identity of exposed amino acids at the points of action appeared to determine which disassembly mechanism was dominant. Whether similar disassembly mechanisms apply to the more heterogeneous and less ordered structures of amorphous aggregate particles remains unclear. In recent experiments with a bacterial bi-chaperone disaggregase, we characterized the rapid disassembly of two physically distinct types of RuBisCO amorphous aggregate using a free solution, single particle fluorescence burst technique known as burst analysis spectroscopy (BAS) [57,113]. We found that, surprisingly, the rate of aggregate disassembly only weakly correlates with particle size over a two-order-of-magnitude range of particle sizes (between 20 and 2000 monomers), suggesting that (1) the internal structure of an aggregate particle is more important than its size and (2) cooperative disassembly and/or fragmentation may also play an important role in amorphous aggregate disaggregation [113]. Other evidence suggests that Hsp70s may play important mechanistic roles beyond Hsp100 recruitment for the bi-chaperone disaggregases. In our examination of amorphous RuBisCO aggregates, we found that brief pre-incubation of aggregates with the bacterial Hsp70 system resulted in structural remodeling of the aggregate particles, but little disassembly [113]. However, pre-incubation resulted in substantially accelerated disaggregation upon subsequent addition of the bacterial Hsp100, compared with experiments in which all the components of the bi-chaperone machinery were added simultaneously [113]. Structural remodeling of aggregates by Hsp70s has also been suggested to occur with amorphous luciferase aggregates and was found to enhance amyloid fibril disassembly [46,49,139].

## Current challenges

**Figure 3:**
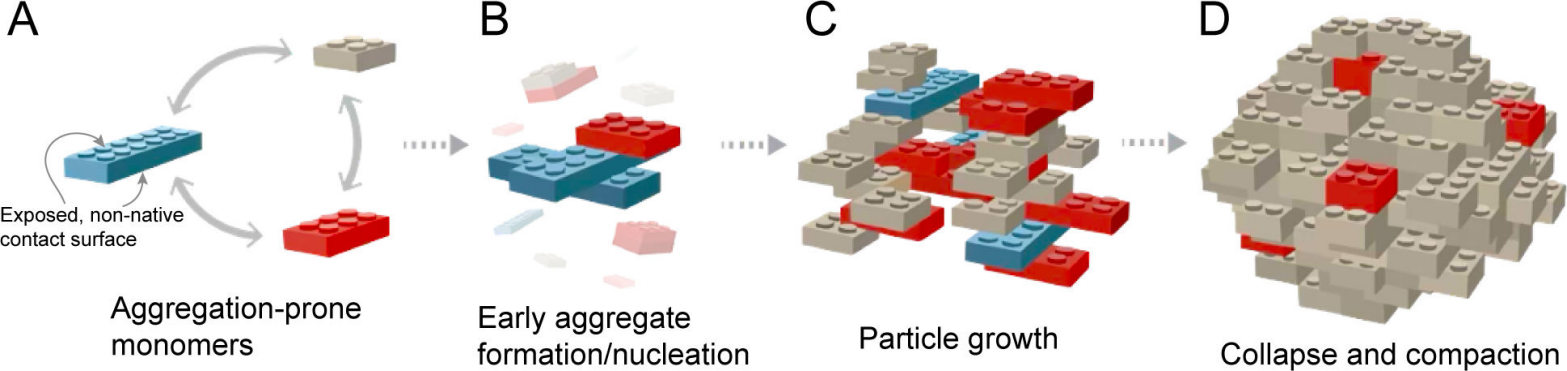
A simplified model of protein aggregate formation, growth, and maturation (**A**) Aggregation-prone folding intermediates are depicted as simple construction blocks. The most expanded and least ordered intermediates are colored blue, somewhat more structured monomers are colored red, and monomers with the most internal structure are colored gray. The upper and lower surface area of each block is proportional to the amount of non-native interaction surface exposed by each monomer. Interconversion between more and less expanded conformations is presumed to be an intrinsic feature of these non-native monomers (indicated by double-headed arrows). (**B**) Above a critical concentration, low-order assemblies form as non-native interaction surfaces on different monomers bind to one another. Early assembly is typically dominated by highly heterogeneous, relatively weak, and poorly organized subunit interactions that can, at least initially, rapidly interconvert. For amyloid-prone proteins (not explicitly illustrated here), low-order oligomer formation supports the appearance of extended β-sheet structures and seed formation. (**C**) Amorphous aggregate particles grow by either addition of monomers to the earliest particles, coalescence of smaller particles into larger aggregate structures, or both. Amorphous aggregates can remain poorly organized and heterogeneous, potentially containing a substantial number of water-filled cavities. However, particle growth can also be accompanied by progressive, internal collapse of the assembled monomers and compaction of the interfaces between them. (**D**) Extensive compaction and interface collapse can produce particles with few surface interaction sites for molecular chaperone engagement and/or that possess internal stabilities that exceed the disruptive impulse a chaperone disaggregase can supply, resulting in aggregate particles that are refractory to disassembly. While amorphous aggregate formation is illustrated here, the more structured growth of amyloid fibrils shares some similar features, including progressive structural compaction and increased molecular chaperone resistance.

It remains unclear whether a common or unified model of protein disaggregation by molecular chaperones exists. The complexity of aggregate particles, formed by potentially hundreds of different cellular proteins, under varying conditions, might require an equally complex array of disassembly mechanisms. However, the fact that so many diverse aggregate species are recognized and, at least most of the time, dismantled by a small subset of molecular chaperones suggests that common aggregate properties, which constrain their disassembly, likely exist and are targeted by the disaggregase chaperones. Identifying the nature of these key features will undoubtedly require more than one approach. One idea is to develop simplified physical models that, at least to begin with, reduce the complexity of aggregate formation and disassembly to fewer, easier-to-handle dimensions. This is essentially the same approach used to develop simple computational lattice models for studying protein folding and amyloid formation [140–144]. Following similar logic, we imagine representing a non-native, aggregation-prone monomer as a toy construction block (Figure 3A). In such a model, the size of a block is inversely proportional to the internal compaction of the monomer, so that smaller blocks expose less external interaction surface, while larger blocks present more. As the interaction surfaces of the monomer blocks begin to semi-randomly stick to one another, loose, heterogeneous, and disordered agglomerations of monomers form (Figure 3B). Over time, the addition of monomers and/or accretion of low-order assemblies leads to particle growth into larger assemblies. At least initially, the poor organization of these early intermediates provides ample exposed surface for ongoing growth, recognition sites for disaggregase chaperone binding, and weaker inter-subunit contacts that are more readily disrupted (Figure 3C). As aggregate particles grow, both monomer and interfacial compaction increase the average particle density, lowering internal hydration and reducing the number and area of exposed attack surfaces for disaggregases (Figure 3D). While this toy model is only one of many that are possible (see for example, [145,146]), it recapitulates many of the features we observe in the disaggregation of RuBisCO and PepQ amorphous aggregates by a bacterial bi-chaperone disaggregase [104,113].

In the end, a mechanistic understanding of protein disaggregation, and how it is affected by aggregate structure, will require solutions to several experimental challenges. First, the heterogeneity of aggregate assemblies, particularly amorphous aggregates, renders them refractory to analysis by traditional biophysical and structural tools alone, which can only capture their mean behavior. Several decades of single molecule and single particle studies have shown that complex biological phenomena frequently cannot be adequately described by their average behavior alone [147,148]. Second, even slight changes in environmental or experimental conditions, such as pH, buffer composition, temperature, and protein concentration, can dramatically alter the properties of both amorphous and amyloid aggregates. Our studies of amorphous RuBisCO aggregates provide one recent example [113], while similar behavior has been noted for a number of other model proteins [50,149–151]. Studies of ⍺-synuclein highlight the polymorphic complexity that bedevils analysis of amyloid aggregates. Different ⍺-synuclein fibril structures have been observed in samples incubated under varying experimental conditions [152,153]. Importantly, fibrils isolated from the brains of multiple system atrophy patients display at least two distinct filament types, and these patient-derived fibrils are structurally distinct from those grown from pure recombinant protein in the lab [153]. Similar structural polymorphisms have been characterized for several other amyloid-forming proteins and, as noted above, appear to contribute to the phenomenon of prion strains [50,54]. Third, the assemblies formed by aggregating proteins are rarely static, but they can continue to change over a wide range of time scales, from seconds to days, even in the absence of ongoing particle growth [44,113,154,155]. This additional layer of kinetic complexity places limits on aggregate sample handling and can constrain the design of experimental systems for studying protein disaggregation.

Testing models of aggregate disassembly by molecular chaperones, such as the toy model we outline above, will likely require a combination of experimental and theoretical approaches. For example, biophysical tools, including chromatography, light scattering, atomic force microscopy, centrifugation sedimentation, circular dichroism, nuclear magnetic resonance spectroscopy, X-ray crystallography, fluorescence spectroscopy, infrared spectroscopy, surface plasmon resonance, and Raman spectroscopy, have all contributed key insights into the average properties of different aggregation phenomena [23,50,114,153,156–162]. By combining these insights with the species-resolving power available from single molecule and single particle tools like fluorescence correlation spectroscopy [163], single molecule Förster resonance energy transfer [164], BAS [113,165], tip-enhanced Raman and infrared spectroscopy [166,167], force spectroscopy [123], native ion mass spectrometry with ultra-high mass detectors [168,169], and single particle cryo-electron microscopy and tomography [153], it seems likely that novel insights into the essential physical properties of different aggregate forms will become available. By incorporating this experimental detail into theoretical models that can be attacked with new theoretical, computational, and machine learning tools [23,170,171], the potential to overcome the daunting complexity of protein aggregation and disaggregation seems more in reach than ever before.

**Perspectives**

- Protein misfolding and aggregation are deep problems in cellular biology that affect the basic physiology of all living organisms and fundamentally constrain the environments in which they are able to survive. Protein aggregates can populate an enormous variety of assembled states that display a wide range of physical properties.
- Cellular proteostasis systems, which include several families of molecular chaperones, are designed to identify, remodel, and dismantle protein aggregates, though some aggregates resist disaggregation. How aggregate structure affects the mechanism of aggregate particle assembly, chaperone resistance, and disease is poorly understood.
- Understanding the link between aggregate structure and chaperone-mediated disaggregation is challenging due to aggregate heterogeneity, structural complexity, and dynamic assembly behaviors and will require the application of several different approaches to untangle.

## Abbreviations

ACD: α-crystallin domain
BAS: burst analysis spectroscopy
CTD: C-terminal domain
LLPS: liquid–liquid phase separation
NBD: nucleotide-binding domain
NEFs: nucleotide exchange factors
NTD: N-terminal domain
SBD: substrate-binding domain
sHsp: small heat shock proteins
